# Design and Preliminary Phantom Study of a 3D-Printed Wrist Immobilization Device for Lateral Radiography

**DOI:** 10.3390/diagnostics16142173

**Published:** 2026-07-12

**Authors:** Natchayaporn Thonapan, Luckika Panthiya, Khamolchanok Khunla, Sukrit Techawattanakijkul, Sarawut Lapmanee

**Affiliations:** 1 Department of Radiological Technology, Faculty of Allied Health Sciences, Thammasat University, Pathum Thani 12120, Thailand; natchayaporn.t@allied.tu.ac.th (N.T.); luckika.p@allied.tu.ac.th (L.P.); khamolchanok.khunla.research@gmail.com (K.K.); sukrit.techawattanakijkul@gmail.com (S.T.); 2 Chulabhorn International College of Medicine, Thammasat University, Pathum Thani 12120, Thailand

**Keywords:** wrist fracture, wrist X-ray, 3D-printed splint, positioning aid, image quality

## Abstract

**Background/Objectives**: Distal radius and carpal fractures are common injuries in emergency radiology, where accurate lateral wrist positioning is essential for diagnostic image quality. Maintaining a true lateral position can be challenging, often requiring manual support and increasing occupational radiation exposure. This pilot phantom study aimed to design, fabricate, and evaluate a novel 3D-printed wrist immobilization device and compare its performance with adhesive tape and sandbag stabilization. **Methods**: A wrist immobilization device was designed using computer-aided design software and fabricated by fused deposition modeling with polylactic acid. A wrist phantom was imaged using three stabilization techniques: the 3D-printed device, adhesive tape, and sandbag support. Image quality was independently assessed by two blinded observers using an eight-point scoring system. Inter-rater reliability was evaluated using the intraclass correlation coefficient (ICC). Positioning time was recorded, and radiopacity was assessed using pixel intensity measurements within predefined regions of interest. **Results**: No significant differences in image quality were observed among the three stabilization techniques (*p* = 0.263). Inter-rater reliability was excellent (ICC = 0.877). Positioning time differed significantly among techniques (*p* = 0.036), with sandbag stabilization requiring the shortest time. Radiopacity analysis demonstrated minimal attenuation (8.6–20.8%) in regions adjacent to the anatomical area of interest, while higher attenuation was limited to non-diagnostic regions. **Conclusions**: The 3D-printed wrist immobilization device produced image quality comparable to conventional stabilization methods with minimal radiographic interference. These findings support its feasibility as a low-cost positioning aid and warrant further evaluation in larger clinical studies.

## 1. Introduction

Plain radiography of the wrist remains one of the most frequently performed skeletal examinations in diagnostic imaging, driven largely by the high incidence of distal radius fractures and the clinical necessity to evaluate a wide spectrum of carpal pathologies [1,2]. Distal radius fractures alone account for 15–21% of all fractures presenting to emergency departments worldwide, representing a substantial diagnostic workload and reinforcing the importance of accurate and reproducible imaging techniques [3].

Standard wrist radiographic assessment relies on orthogonal projections, of which the lateral view plays a critical role in evaluating the radiocarpal and distal radioulnar joint relationships, assessing carpal alignment, and determining the fracture angulation [4]. A true lateral position is achieved when the distal radius and ulna are appropriately superimposed, with correct alignment of the carpal structures [5,6]. Even minimal rotational deviation of the forearm may alter these anatomical relationships, potentially obscuring subtle fractures or affecting assessment of displacement, thereby influencing clinical decision-making and treatment planning [4,7,8,9].

In routine clinical practice, achieving and maintaining this strict positioning is often challenging. Patients presenting with acute trauma frequently experience significant pain, swelling, or mechanical instability, which limits their ability to cooperate or sustain the required neutral wrist posture [10]. Additional challenges arise in vulnerable populations, including elderly patients with reduced mobility, individuals with neurological impairment, and those with altered levels of consciousness [2,7,10]. Under such conditions, radiographers are frequently required to manually support or stabilize the patient’s limb during image acquisition, resulting in occupational exposure to scattered ionizing radiation [11].

Although the radiation dose per exposure is relatively low, repeat imaging due to suboptimal positioning may contribute to unnecessary cumulative radiation exposure to the radiographer, workflow inefficiency, and patient discomfort [12,13,14,15]. Furthermore, reliance on manual stabilization introduces operator-dependent variability, potentially reducing positioning reproducibility [13,14].

Conventional positioning aids, i.e., foam wedges, sandbags, and Velcro straps, are commonly employed to assist in patient stabilization; however, their functional capabilities are inherently limited [16,17]. These tools provide only gross positional support and lack the structural precision required to enforce strict neutral rotation of the wrist and forearm. Moreover, they are not anatomically contoured and cannot adequately accommodate inter-individual variability in wrist size and morphology [16,17,18]. As a result, achieving a true lateral projection may remain inconsistent, particularly in non-cooperative or high-pain scenarios, highlighting the need for more specialized and standardized immobilization solutions [19,20].

In recent years, three-dimensional (3D) printing has emerged as a transformative technology in medicine, enabling the fabrication of customized, cost-effective, and rapidly deployable devices tailored to specific clinical applications [21]. Fused deposition modeling (FDM) was selected for device fabrication due to its accessibility, low cost, and compatibility with polylactic acid (PLA) filament [22]. PLA is easy to fabricate, dimensionally stable, and demonstrates relatively low X-ray attenuation within the diagnostic energy range, making it suitable for applications where radiolucency is required [23,24]. Previous studies have reported the use of 3D-printed materials in the development of tissue-equivalent phantoms, patient-specific bolus for radiotherapy, and immobilization devices, supporting its utility in precision medical applications [23,25,26]. The ability to iteratively modify digital designs and rapidly fabricate prototypes further enhances its suitability for point-of-care innovation within radiology departments [21].

Despite these advances, there remains a notable lack of dedicated 3D-printed devices specifically engineered for wrist positioning in lateral radiography [27]. The existing literature has predominantly focused on larger anatomical regions or radiotherapy applications, with limited attention given to the unique geometric and biomechanical constraints associated with small-joint imaging [28]. The absence of purpose-built immobilization tools for this application represents an unmet clinical need, particularly in settings where patient cooperation is limited and imaging throughput is high. Addressing this gap may improve both image quality and occupational safety while reducing repeat imaging rates [29].

Accordingly, the present study was undertaken with two primary objectives: (i) to design and fabricate a parametric wrist immobilization device using PLA-based FDM technology, specifically optimized to facilitate accurate lateral positioning across a range of adult wrist anatomies; and (ii) to conduct a structured phantom-based evaluation of the device, assessing its radiolucency, impact on image quality, and positioning reproducibility using quantitative metrics. This preliminary investigation aims to establish foundational evidence supporting the feasibility of the proposed device and to provide a basis for subsequent validation in clinical settings.

## 2. Materials and Methods

### 2.1. Device Design

The wrist immobilization device was designed using Shapr3D parametric computer-aided design software version 26.40 (Shapr3D Zrt., Budapest, Hungary) on an iPad Air (gen3) platform. The design objective was to securely maintain the wrist in a true lateral position (ulnar aspect down) directly on the flat panel detector, while allowing adaptability to different wrist sizes.

**Figure 1:**
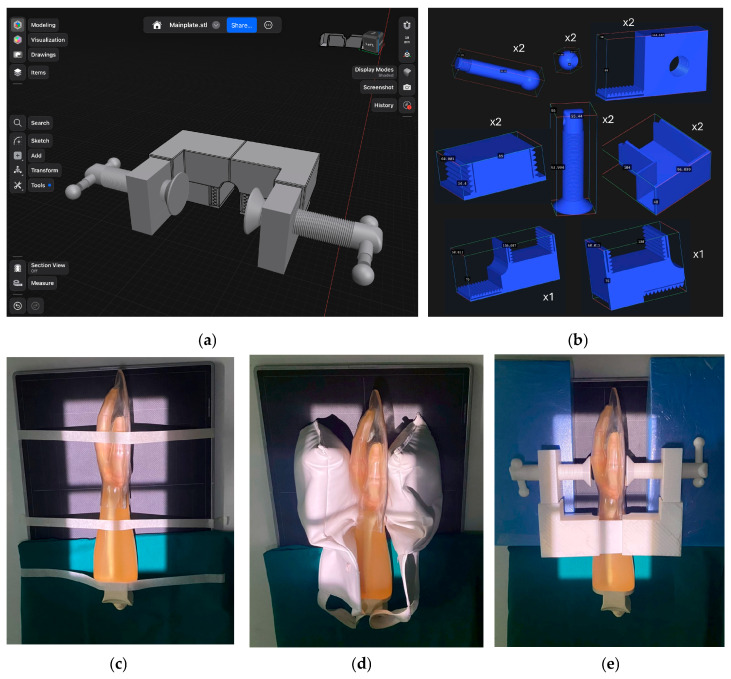
Design and components of the 3D-printed wrist immobilization device and positioning methods. (**a**) Three-dimensional model of the assembled device illustrating the overall structure, including the base platform, dorsal support, and adjustable clamping system. (**b**) Disassembled view of the device components showing individual part designs and their respective measurements for fabrication. Positioning of the forearm phantom for true lateral radiography using three methods: (**c**) adhesive tape fixation, (**d**) sandbag support, and (**e**) 3D-printed wrist immobilization device.

The device comprised 14 individual components, which were incorporated into three principal components. First, a fixed dorsal backstop was positioned perpendicular to the detector surface to ensure consistent alignment of the forearm. Second, two opposing clamping arms were integrated to stabilize the wrist; these arms were adjustable via a threaded screw mechanism, enabling controlled compression without excessive pressure. Third, a low-profile base was designed to minimize interference with the imaging field and to avoid overlap with the region of interest (ROI), as shown in Figure 1.

The threaded screw system was generated using a revolve function to create a three-dimensional helical thread, followed by Boolean subtraction to produce the corresponding internal nut geometry. Anatomically conforming surfaces were created using spline-based curves and mirrored symmetrically to ensure balanced support. The finalized design was exported in stereolithography (STL) format for subsequent slicing and fabrication.

### 2.2. Device Fabrication

The STL model was processed using Bambu Studio slicing software version 2.7.1 and fabricated with a Bambu Lab X1E FDM 3D printer (Bambu Lab, Shenzhen, China). Printing parameters were optimized for structural integrity and dimensional accuracy and are summarized in Table 1.

White PLA filament (1.75 mm diameter) was selected due to its relatively low radiographic attenuation, print stability, low cost, and ease of post-processing. Automatic support structures were generated for overhanging regions to ensure print fidelity. Following fabrication, support materials were carefully removed, and all contact surfaces were lightly sanded to eliminate rough edges and improve safety and comfort during use.

### 2.3. Phantom and Imaging Equipment

All experiments were performed using the upper-limb segment of a Whole Body Tissue-Equivalent Phantom PBU-60 (Kyoto Kagaku Co., Ltd., Kyoto, Japan), which replicates the X-ray attenuation properties of human soft tissue and bone. Image acquisition was conducted using a Fujifilm FDR Smart X general radiographic system (Fujifilm Corporation, Tokyo, Japan), equipped with a Fujifilm FDR D-EVO II DR-ID 1200 flat panel detector (14 × 17 inches, 150 μm pixel pitch, 16-bit dynamic range).

Exposure parameters were standardized across all acquisitions at 70 kVp, 125 mA, and 40 ms (5 mAs). The source-to-image distance was fixed at 100 cm, in accordance with the routine wrist radiography protocol employed at the Faculty of Allied Health Sciences, Thammasat University.

### 2.4. Experimental Protocol

The forearm phantom was positioned using three different techniques: adhesive tape fixation, sandbag support, and 3D-printed wrist immobilization device. For the adhesive tape condition, the forearm was secured using adhesive tape applied at the distal, midshaft, and proximal segments to minimize movement, while avoiding coverage of the wrist joint. In the sandbag condition, sandbags were placed along both sides of the phantom to stabilize the position. For the 3D-printed device condition, the forearm was immobilized within a custom-designed frame equipped with adjustable clamps to stabilize both the forearm and palm, while avoiding obstruction of the wrist joint. Foam supports were applied laterally to compensate for the height discrepancy between the 3D-printed device and the phantom. This adjustment contributed to improved positioning stability and reproducibility across repeated trials (Figure 1). Positioning was performed independently by two operators. Each operator performed five repeated imaging trials for each positioning method, resulting in 10 radiographs per technique. A total of 30 radiographs were acquired and analyzed. The acquisition sequence was fixed rather than randomized and was performed consistently across all repeated trials. In all conditions, operators were instructed to achieve a true lateral wrist projection.

The central ray was directed perpendicular to the image receptor and centered at the level of radiocarpal joint. For each trial, the phantom was completely removed and repositioned within the device without altering the device configuration, allowing assessment of positioning reproducibility. All images were transferred to the Synapse PACS workstation (Fujifilm, Tokyo, Japan) for subsequent analysis. To assess workflow efficiency, positioning time was recorded in seconds from the initiation of positioning to the completion of final alignment prior to radiographic exposure.

### 2.5. Image Quality Assessment

Each radiograph was independently assessed by two radiologic technology instructors using a structured image quality scoring system (Table 2). Image quality was assessed using an 8-point scoring system developed for the present study based on published lateral wrist radiographic quality criteria described by Barot et al. (2025) [30]. Four image quality criteria were evaluated, namely, distal radius–ulna superimposition, metacarpal superimposition, carpal alignment, and motion artifact. Each criterion was scored on a scale of 0–2, yielding a maximum total score of 8. For the purpose of the study, total scores were interpreted as follows: 7–8 indicated excellent image quality consistent with a true lateral projection, 5–6 indicated good image quality, 3–4 indicated fair image quality, and 0–2 indicated poor image quality. The final image quality score for each radiograph was calculated as the mean of the scores assigned independently by the two evaluators. Higher scores indicated better compliance with true lateral wrist positioning criteria and overall image quality.

Radiographs were presented in randomized order prior to evaluation. Although the evaluators performed independent assessments, complete blinding was not feasible because certain positioning methods could potentially be identified from the radiographs.

### 2.6. Repeatability and Inter-Rater Reproducibility Analysis

Positioning repeatability was evaluated using the coefficient of variation (CV) of total image quality scores obtained from five repeated acquisitions for each technique. The CV provides a normalized measure of dispersion relative to the mean, allowing comparison of variability across measurements.

$$CV\;\left(\%\right)=\left(\frac{SD}{\bar{x}}\right)\times 100$$

where SD represents the standard deviation and $\bar{x}$ denotes the arithmetic mean of the scores. The CV was interpreted according to predefined thresholds: values < 10% indicate excellent repeatability, 10–20% indicate good or acceptable repeatability, 20–30% indicate moderate repeatability, and values > 30% indicate poor repeatability.

Inter-rater reproducibility of image quality scores between two evaluators was assessed using the intraclass correlation coefficient (ICC), based on a two-way random-effects model with absolute agreement (ICC [2, 1]) and corresponding 95% confidence intervals (CIs). ICC values were interpreted as poor (<0.50), moderate (0.50–0.75), good (0.75–0.90), and excellent (>0.90), according to established guidelines.

### 2.7. Radiolucency Evaluation

The radiolucency of the 3D-printed device was quantitatively evaluated by measuring pixel intensity values (arbitrary units, AU) from five ROIs on a representative radiographic image using Synapse PACS. The selected ROIs included: (1) background (air), (2) the thick edge of the clamping arm, (3) the body of clamping arm, (4) the lateral part of forearm fixation, and (5) the middle extendable part of the forearm fixation. Mean pixel intensity is directly proportional to transmitted X-ray fluence; therefore, higher pixel values correspond to lower attenuation and greater radiolucency. To standardize comparisons, the percentage difference in pixel intensity relative to the background was calculated using the following relationship:

%Difference = [(I_background − I_device)/I_background] × 100

All measurements were performed across five repeated acquisitions, and the resulting values were expressed as mean ± standard deviation (SD) to minimize random variation and improve measurement reliability.

### 2.8. Statistical Analysis

Statistical analyses were performed using IBM SPSS Statistics version 31.0 (IBM Corp., Armonk, NY, USA). Non-parametric statistical methods were selected because image quality scores were derived from an ordinal scoring system and the pilot study involved a relatively small sample size. Image quality scores and positioning time among the three positioning techniques were compared using the Kruskal–Wallis test. Post hoc pairwise comparisons were subsequently performed using the Mann–Whitney U test where appropriate. A *p*-value < 0.05 was considered statistically significant.

## 3. Results

### 3.1. Fabricated Device Characteristics 

**Figure 2:**
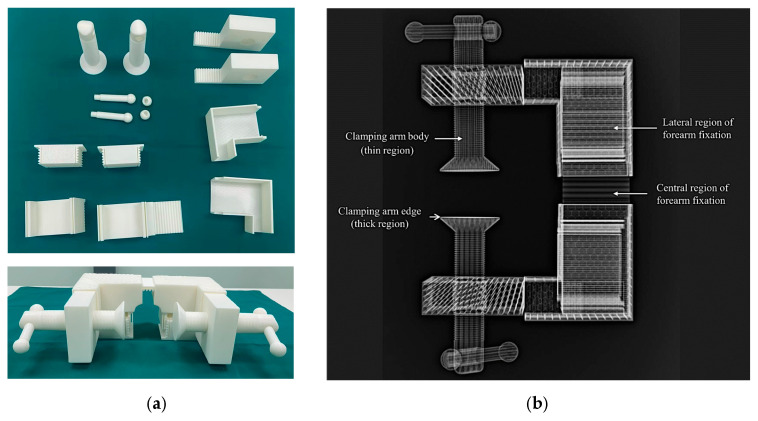
Fabricated 3D-printed wrist immobilization device. (**a**) Disassembled view showing individual separated components and the fully assembled device illustrating its overall structural configuration. (**b**) Radiographic appearance of the 3D-printed wrist immobilization device, demonstrating differential material density across the assembled structure, including the clamping arm edge (thick contact region), the clamping arm body (thin region), and the lateral and central sections of the forearm fixation unit.

The positioning device was successfully fabricated using additive manufacturing and subsequently assembled without structural complications. The final construct comprised 14 discrete components that were assembled into a functional positioning device. Two adjustable opposing lateral clamping arms were positioned at the palmar region and connected to a centrally positioned threaded screw mechanism, enabling controlled adjustment of jaw width over a 50 mm range. The forearm fixation part provided adjustable width, ranging from 30 to 70 mm, and adjustable height, ranging from 50 to 75 mm, allowing adaptability across commonly encountered forearm dimensions (Figure 2a).

The total mass of the fabricated device was approximately 710 g, reflecting a balance between structural integrity and practical handling requirements. The device incorporated an internal hexagonal infill architecture, with thinner lateral walls of the lattice oriented along the X-ray beam path during imaging.

### 3.2. Radiolucency

The radiographic image of the 3D-printed wrist immobilization device is shown in Figure 2b. Quantitative assessment of radiolucency was performed using pixel intensity analysis, with results summarized in Table 3. The background region (air) demonstrated a mean pixel intensity of 2650 AU, serving as the reference for maximal X-ray transmission.

The thick outer edge of the clamping arm exhibited a mean pixel intensity of 685 AU, corresponding to a 74.16% reduction relative to the background. The lateral region of forearm fixation showed a mean pixel intensity of 2100 AU, demonstrating a 20.77% reduction relative to the background.

In contrast, the thin clamping arm body region yielded a mean pixel intensity of 2257 AU, representing a 14.83% reduction relative to the background. In addition, the central thin region of the extendable forearm fixation component exhibited a mean intensity of 2423.6 AU, representing only an 8.56% reduction relative to the background.

### 3.3. Positioning Reproducibility and Image Quality

**Figure 3:**
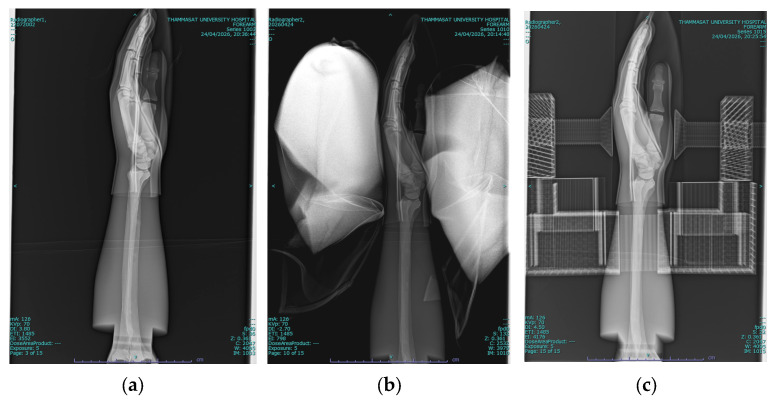
Representative radiographic images from three different positioning techniques. (**a**) Adhesive tape fixation technique. (**b**) Sandbag support technique. (**c**) 3D-printed wrist immobilization device.

The radiographic images of three positioning techniques are demonstrated in Figure 3. The mean quality scores from the two evaluators are shown in Table 4. All positioning techniques showed good image quality, with adhesive tape fixation demonstrating the highest mean image quality score (6.8 ± 1.06), followed by the 3D-printed device (6.65 ± 0.91), and sandbag support (6.1 ± 0.97). No significant difference in image quality scores was observed among the three positioning techniques (Kruskal–Wallis test: H = 2.675, *p* = 0.263). Positioning repeatability from five repeated acquisitions for each technique is shown as CV. The CV was 15.58% for adhesive tape fixation, 15.84% for sandbag support, and 13.75% for the 3D-printed device, indicating good repeatability across three techniques.

Details of inter-rater reproducibility are presented in Table 5. When analyzed by positioning technique, the ICC value was 0.92 for adhesive tape fixation, 0.90 for sandbag support, and 0.83 for the 3D-printed wrist immobilization device. These findings indicate that the scoring results showed good-to-excellent inter-rater agreement across the three positioning techniques.

### 3.4. Positioning Time Analysis

A comparison of setup time among the three positioning techniques is shown in Table 6. Positioning time differed significantly among the three positioning techniques (Kruskal–Wallis test: H = 6.637; *p* = 0.036). Post hoc pairwise comparisons using the Mann–Whitney U test demonstrated significantly shorter positioning time for sandbag support compared with adhesive tape fixation (*p* = 0.021) and the 3D-printed device (*p* = 0.041). No significant difference was observed between adhesive tape fixation and the 3D-printed device, as shown in Table 7.

## 4. Discussion

This study provides proof-of-concept evidence that a purpose-designed, FDM-fabricated PLA wrist immobilization device can reliably maintain a true lateral wrist position during radiographic acquisition in a controlled phantom setting. The 3D-printed device achieved a mean image quality score of 6.65/8, which approaches the true lateral positioning category and indicates that the device consistently meets the geometric criteria required for diagnostic lateral wrist imaging—namely, complete superimposition of the distal radius and ulna, appropriate metacarpal overlap, correct carpal alignment, and absence of motion artifacts. These findings align with prior reports on 3D-printed positioning aids in comparable anatomical regions and support the design principle of separating load-bearing (radiodense) components from the anatomical ROI [31,32].

Accurate true lateral wrist positioning is clinically important because it enables reliable assessment of distal radius alignment, carpal relationships, and distal radioulnar joint congruency [30]. Even small degrees of forearm malrotation can alter the apparent anatomical relationships of the distal radius, ulna, and carpal bones, potentially affecting the interpretation of fractures, joint alignment, and carpal instability [33]. Consequently, suboptimal positioning may result in non-diagnostic or clinically misleading radiographs, necessitating repeat imaging and potentially delaying diagnosis and treatment. Although wrist radiography is associated with relatively low radiation exposure, repeat examinations increase cumulative patient dose, consume additional staff time, and reduce departmental workflow efficiency. Therefore, positioning aids capable of consistently reproducing true lateral wrist projections may improve diagnostic confidence, reduce repeat imaging rates, and support radiation protection principles.

The radiolucency results provide a clear mechanistic explanation for this performance. The thin clamping arm demonstrated only a 14.83% reduction in pixel intensity relative to background (2257.4 vs. 2650.6 AU), indicating minimal X-ray attenuation and effective radiographic transparency under diagnostic conditions. This is consistent with the known half-value layer characteristics of PLA, where thin sections attenuate only a small fraction of incident X-ray fluence [27]. In contrast, the thicker outer frame exhibited substantial attenuation (74.16%), as expected from increased material thickness. Importantly, this radiodense region was intentionally positioned outside the imaging field, ensuring that it does not obscure clinically relevant anatomy. This spatial segregation strategy represents a key design strength and is broadly applicable to the development of radiolucent positioning devices for other joints [34]. Furthermore, the radiolucency analysis in the present pilot study was intended as a descriptive evaluation of attenuation characteristics rather than a formal inferential comparison between structural regions.

Positioning reproducibility, quantified by a CV of 13.75%, falls within the Good/Acceptable range and is comparable to values reported in the literature for 3D-printed immobilization systems used in radiotherapy, where reduction CVs have been observed [35,36,37]. The lower-scoring acquisition was attributable to minor rotational misalignment of the forearm during repositioning, leading to incomplete radius–ulna superimposition and suboptimal metacarpal overlap. This indicates a specific limitation of the current device design: while the adjustable clamping mechanism effectively constrains mediolateral movement, it does not adequately prevent axial rotation (pronation/supination). As a result, small variations in operator handling may introduce rotational error sufficient to affect image quality.

Furthermore, the incorporation of anatomical alignment landmarks, forearm contour supports, or rotational locking mechanisms may further reduce operator-dependent variability and improve positioning consistency across repeated examinations. Such refinements could be particularly beneficial when imaging patients with differing forearm morphology or limited ability to maintain a stable position. By minimizing residual pronation–supination movement, future device designs may further enhance reproducibility and image quality, thereby improving the reliability of true lateral wrist radiographs.

Several limitations should be considered. First, this study was conducted using a single wrist phantom, which limited anatomical variability and may have reduced the generalizability of the findings to clinical patient populations. Although two operators participated in the positioning procedures, the study design did not fully capture the broad range of operator-dependent variation encountered in routine clinical practice. In addition, positioning accuracy was assessed using established radiographic quality criteria rather than direct quantitative measurements of angular deviation. While these criteria are commonly used to evaluate true lateral wrist positioning in clinical practice, they do not provide an objective measure of rotational error. Future studies should include multiple operators, a more diverse sample representing a wider range of wrist anatomies and clinical conditions, and quantitative angular measurements to further validate positioning accuracy and device reproducibility. Studies with larger sample sizes may additionally benefit from more advanced repeated-measures statistical analyses to further evaluate positioning reproducibility and operator-related variability. Separate evaluation of intra-operator repeatability may also provide additional insight into device consistency and reproducibility.

Second, image quality evaluation was performed by two observers, and inter-rater reliability analysis demonstrated good agreement. However, complete blinding of the evaluators was not feasible because the positioning characteristics and fixation methods were visible on the radiographs, which may have introduced observer bias [38].

Third, the use of low-density (15%) hexagonal infill, while beneficial for radiolucency, may reduce structural stiffness. Under clinical loading conditions, increased mechanical stress from patient movement could potentially result in structure deformation. Future studies may further evaluate the structural stability of the device under practical operating conditions.

Fourth, although the device improved positioning accuracy, its setup time was significantly longer than that required for conventional sandbag stabilization. This additional workflow requirement may limit practicality in high-volume clinical environments such as emergency departments, where rapid patient throughput is essential. However, the current prototype was evaluated during an early stage of development, and the recorded setup times may partially reflect operator unfamiliarity with the device. Future optimization through ergonomic redesign, simplified assembly, rapid-locking mechanisms, and standardized training protocols may reduce preparation time while maintaining positioning accuracy and reproducibility.

Fifth, the relatively low friction coefficient of PLA contributed to device slippage on the detector surface, suggesting the need for integrated anti-slip base materials, such as vulcanized rubber or silicone pads, in future designs. Finally, although the current adjustment range was designed to accommodate commonly encountered forearm dimensions, it may not fully support all patient groups, particularly pediatric or large-frame individuals, indicating the need for scalable or parametric design modifications. Clinical validation studies involving real patients are warranted to confirm the effectiveness, usability, and safety of the device in routine radiographic practice.

Future work should proceed along both technical and clinical pathways. From a technical perspective, optimization of infill geometry and material selection, e.g., polyethylene terephthalate glycol for enhanced stiffness or thermoplastic polyurethane for improved compliance), should be explored to balance radiolucency, durability, and patient comfort [39]. Integration of anti-rotation features and surface modifications to improve stability are also recommended. From a clinical standpoint, prospective studies involving multiple operators and patients are needed to evaluate usability, patient comfort (especially in acute injury settings), and the potential to reduce repeat imaging and occupational radiation exposure. Additionally, the modular design framework demonstrated here may be extended to other anatomical regions requiring precise positioning, such as the lateral ankle or elbow, thereby broadening its clinical applicability [40,41].

The findings in the present study support the feasibility of using low-cost, 3D-printed PLA devices to improve positioning accuracy in musculoskeletal radiography, while also identifying clear pathways for refinement and clinical translation.

## 5. Conclusions

A parametric, FDM-fabricated PLA wrist immobilization device was successfully developed and validated in a tissue-equivalent phantom, demonstrating consistent production of high-quality near-true-lateral wrist radiographs with acceptable positioning reproducibility. The device exhibited clinically negligible X-ray attenuation within the anatomical region of interest, while structurally necessary radiodense components were deliberately positioned outside the imaging field to prevent interference with diagnostic visualization. These findings establish a clear design–performance framework for radiolucent positioning devices and support progression toward clinical validation. As a low-cost, customizable solution, this device has strong potential to reduce occupational radiation exposure during lateral wrist radiography, particularly in non-cooperative patients, without compromising diagnostic image quality.

## Figures and Tables

**Table 1:** Fabrication parameters for 3D-printed wrist immobilization device.

Parameter	Value
Printer	Bambu Lab X1E
Material	PLA (white, 1.75 mm)
Layer height	0.2 mm
Infill density	15%
Infill pattern	Hexagon (honeycomb)
Wall count	2
Supports	Enabled (tree supports at overhangs)
Nozzle temperature	220 °C
Bed temperature	60 °C

**Table 2:** Image quality scoring rubric for lateral wrist radiography.

Criterion	Scoring Description	Max Score
Distal radius–ulna superimposition	2 = complete overlap; 1 = partial; 0 = no overlap.	2
Metacarpal superimposition	2 = complete overlap; 1 = partial; 0 = separated.	2
Carpal alignment (lunate–capitate; pisiform–scaphoid)	2 = excellent alignment; 1 = minor deviation; 0 = clear deviation.	2
Motion artifact/image stability	2 = no motion artifact; 1 = minor; 0 = obvious.	2

**Table 3:** Pixel intensity measurements and percentage differences relative to background.

Location	Measurement (*n*)	Mean Pixel Intensity ± SD (AU)	Difference vs. Background (%)
Background	5 (2268, 2618, 2726, 2592, 2649)	2650.6 ± 51.20	0 (reference)
Clamping arm edge (thick region)	5 (603, 532, 550, 796, 944)	685 ± 178.72	74.16%
Clamping arm body (thin region)	5 (2135, 2294, 2362, 2277, 2219)	2257.4 ± 85.32	14.83%
Lateral region of forearm fixation	5 (2067, 2099, 2108, 2249, 1977)	2100 ± 98.09	20.77
Central region of forearm fixation	5 (2468, 2714, 2353, 2204, 2379)	2423.6 ± 188.08	8.56

AU = arbitrary units. Background reference value: 2650.6 AU. % reduction calculated as [(background − region)/background] × 100.

**Table 4:** Image quality and repeatability assessment.

Positioning Technique	*n*	Mean Score ± SD	CV (%)	Repeatability Interpretation
Adhesive tape fixation	10	6.8 ± 1.06	15.58	Good
Sandbag support	10	6.1 ± 0.97	15.84	Good
3D-printed device	10	6.65 ± 0.91	13.75	Good

Kruskal–Wallis test: H = 2.675, *p* = 0.263.

**Table 5:** Inter-rater reproducibility.

Positioning Technique	ICC	95% CI	*p*-Value	Interpretation
Adhesive tape fixation	0.92	0.826–0.978	<0.001	Excellent
Sandbag support	0.90	0.792–0.987	<0.001	Good–Excellent
3D-printed device	0.83	0.641–0.978	<0.001	Good

**Table 6:** Comparison of positioning time among techniques.

Positioning Technique	*n*	Mean (s) ± SD	Range(s)
Adhesive tape fixation	10	112.5 ± 38.56	75–196
Sandbag support	10	73.6 ± 23.11	40–106
3D-printed device	10	119.4 ± 46.50	68–185

Kruskal–Wallis: H = 6.637; *p* = 0.036.

**Table 7:** Post hoc pairwise Mann–Whitney U test results for positioning time.

Comparison	U Statistic	*p*-Value	Interpretation
Tape vs. sandbag	81.0	0.021	Sandbag significantly faster
Tape vs. 3D-printed device	47.0	0.850	No significant difference
Sandbag vs. 3D-printed device	22.5	0.041	Sandbag significantly faster

## Data Availability

The data supporting the findings of this study are available from the corresponding author upon reasonable request.

## Abbreviations

The following abbreviations are used in this manuscript:

3D	Three-dimensional
AU	Arbitrary unit
CV	Coefficient of variation
FDM	Fused deposition modeling
ICC	Intraclass correlation coefficient
PLA	Polylactic acid
ROI	Region of interest
STL	Stereolithography
